# A Case of *Haemophilus influenzae* Colonisation in Bronchiectasis Following COVID‐19: Post‐COVID‐19 Bronchiectasis as a Structural Hotbed for Chronic Airway Infections

**DOI:** 10.1002/rcr2.70287

**Published:** 2025-07-23

**Authors:** Issei Oi, Yuta Okada, Naoki Fujimoto, Saiki Yoshimura, Shogo Toyama, Takanori Ito, Takuma Imakita, Osamu Kanai, Kohei Fujita, Kiminobu Tanizawa

**Affiliations:** ^1^ Division of Respiratory Medicine Center for Respiratory Diseases, National Hospital Organization Kyoto Medical Center Kyoto Japan

**Keywords:** bronchiectasis, chronic respiratory infection, colonisation, COVID‐19, *H. influenzae*

## Abstract

Coronavirus Disease 2019 (COVID‐19) can lead to respiratory sequelae, including bronchiectasis. While bronchiectasis following tuberculosis is a breeding ground for many bacterial infections/colonisation, there are few reports of bronchiectasis following COVID‐19 being a hotbed for bacterial infection/colonisation. We present a case of a 69‐year‐old female who developed bronchiectasis following COVID‐19 pneumonia. The patient had no abnormal findings on chest CT scan for 5 years before COVID‐19. She developed persistent cough and sputum after COVID‐19 and chest CT just after COVID‐19 revealed new bronchiectasis. One year later, she was introduced to our department for repeating exacerbation of chronic respiratory infection, and *Haemophilus influenzae* was detected in sputum. This case highlights the potential for *H. influenzae* to infect/colonise in post‐COVID‐19 bronchiectasis. While there have been few reports of chronic airway infection complicating bronchiectasis after COVID‐19 until now, long‐term respiratory follow‐up and management of bacterial colonisation are crucial in these patients in the future. This case suggests that COVID‐19 can predispose individuals to bacterial infection in the setting of bronchiectasis, emphasising the need for vigilance in post‐COVID‐19 airway management.

## Graphical abstract

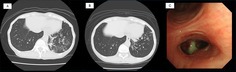

## 1 Introduction

Coronavirus disease 2019 (COVID‐19) is known to cause various respiratory sequelae even after the acute phase of pneumonia. One such sequela is bronchiectasis, with a higher risk observed especially in severe COVID‐19 cases [1]. Bronchiectasis develops against a background of airway infection and inflammation, and bacterial colonisation is known to contribute to the worsening of the condition. Bronchiectasis itself is known to be a hotbed for bacterial infection/colonisation, a condition commonly seen in post‐tuberculosis bronchiectasis. However, there are few reports of secondary bacterial infection/colonisation in bronchiectasis after COVID‐19.

Recently, a case of *Mycobacterium avium complex* (MAC) infection/colonisation in post‐COVID‐19 bronchiectasis has been reported, suggesting that airway structural changes following COVID‐19 may be a risk factor for MAC infection [2]. However, there are no reports on *Haemophilus influenzae* ( *H. influenzae* ) infection/colonisation in post‐COVID‐19 bronchiectasis. *H. influenzae* is a commonly detected pathogen in patients with chronic airway diseases and has been shown to be involved in the exacerbation and progression of bronchiectasis. Here, we report a case of newly developed bronchiectasis with subsequent *H. influenzae* infection/colonisation following COVID‐19.

## 2 Case Report

**FIGURE 1:**
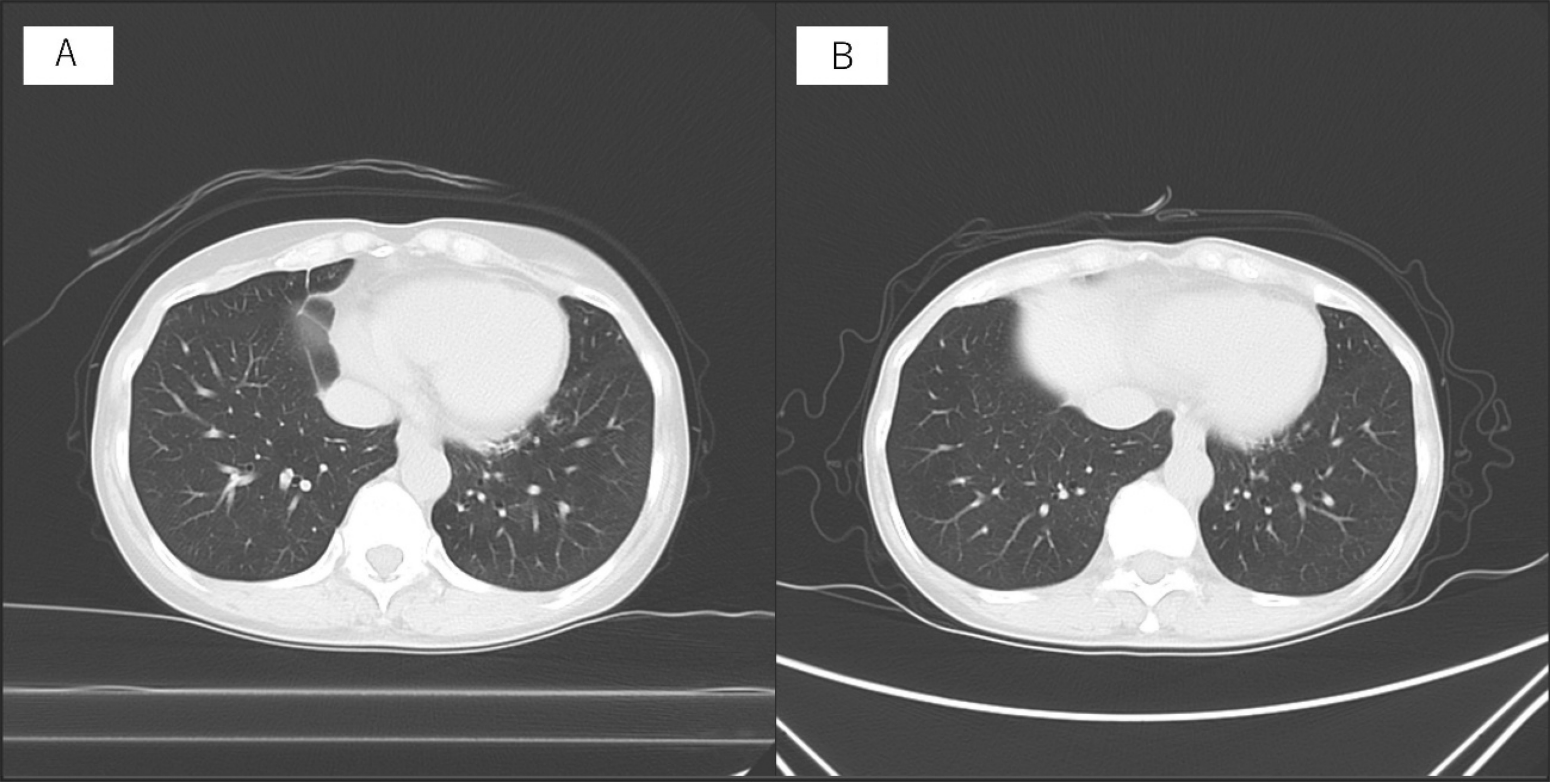
CT scans before COVID‐19. (A) Chest CT scan at 5.5 years before COVID‐19. (B) Chest CT scan at one half year before COVID‐19. No abnormal findings were observed.

The patient was a 69‐year‐old female. She had previously visited our department of respiratory medicine complaining of cough and was treated and followed up for suspected cough variant asthma. Inhaled corticosteroid was administered, and as her symptoms stabilised, follow‐up at our institution was discontinued, and she was referred to a local physician. Chest CT at the initial visit, which was 5 years prior to the end of follow‐up, showed no abnormal shadows (Figure 1A), and the same was true at the end of follow‐up (Figure 1B).

**FIGURE 2:**
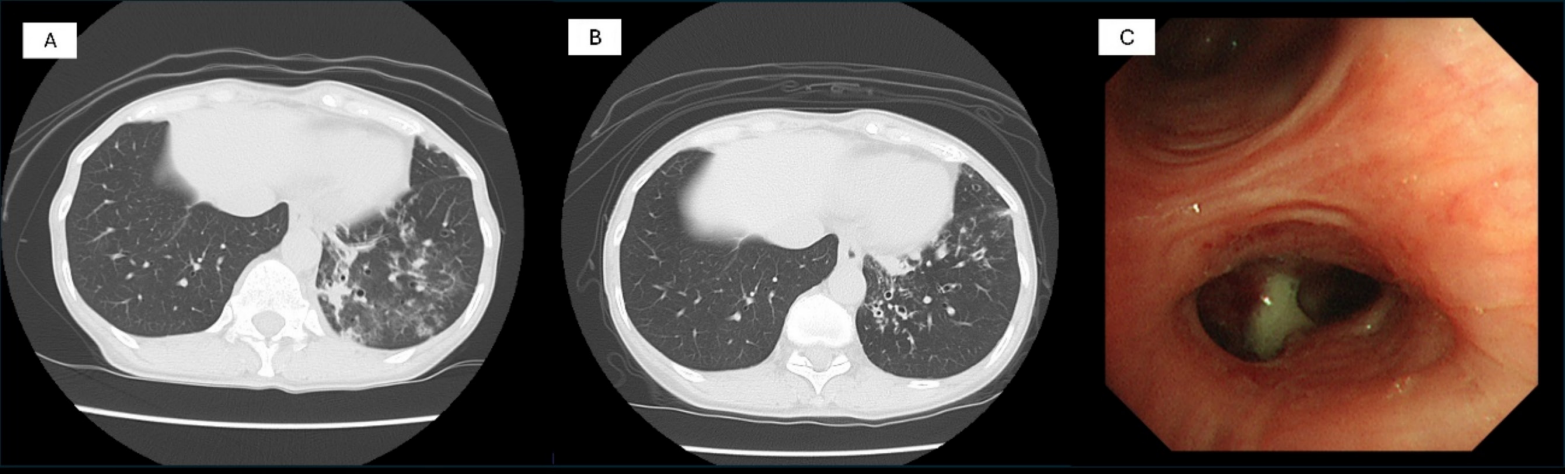
CT scan and bronchoscopy findings at re‐visit at 1 year after COVID‐19. (A) CT scan at 1 month after COVID‐19. New bronchiectasis was observed in her left lower lobe, and new infiltrations around bronchiectasis showed acute exacerbation. (B) Bronchiectasis persisted and acute exacerbation was observed again. (C) Bronchoscopy showed sputum in the left lower bronchus, and *H. influenzae* was detected.

Approximately half a year later, the patient contracted COVID‐19 and was treated for moderate pneumonia. She complained of persistent cough and sputum and was referred to our department again due to recurrent pneumonia 1 year later after COVID‐19. CT scan just after COVID‐19 showed bronchiectasis in her left lower lobe, which was not observed at the previous discharge (Figure 2A). A chest CT scan at the time of the re‐visit revealed developed bronchiectasis in the left lower lobe (Figure 2B). *H. influenzae* was detected in sputum bacterial culture. Bronchoscopy revealed the accumulation of purulent secretions in the lower respiratory tract (Figure 2C), and acid‐fast bacteria or fungi were not detected.

## 3 Discussion

This case highlights the occurrence of residual bronchiectasis after COVID‐19 followed by *H. influenzae* infection.

Several mechanisms are considered to underlie the development of bronchiectasis after COVID‐19. After pneumonia caused by COVID‐19, airway fibrosis and structural destruction occur, leading to impaired mucus clearance and decreased local immune function [1]. Bronchiectasis is a risk factor for airway colonisation by pathogenic microorganisms, but, unlike post‐tuberculosis bronchiectasis, there are few reports of bacterial infection/colonisation in post‐COVID‐19 bronchiectasis. This may be partly because COVID‐19 is a relatively new disease, having emerged only about 5 years ago. Another hypothesis is that COVID‐19 primarily causes inflammation in the interstitial spaces, which differs from the central airways typically affected in chronic airway infections. In fact, chronic airway infections other than fungal infections are rarely associated with traction bronchiectasis in interstitial pneumonia. However, MAC infection in post‐COVID‐19 bronchiectasis has been reported recently [2], suggesting that changes in the airway environment following COVID‐19 may pose a risk for new infections. We may encounter many similar cases in the future.

In this case, bronchiectasis was identified following persistent cough and sputum production after recovery from COVID‐19, and *H. influenzae* was detected. *H. influenzae* tends to colonise, especially in patients with chronic respiratory diseases with impaired airway defence mechanisms, and has been shown to be associated with the expansion of radiological lesions and an increase in the frequency of mild exacerbations [3]. It has also been reported that chronic infection can contribute to the progression of bronchiectasis. No bronchiectasis was observed until the time of discharge for 5 years, so COVID‐19 is considered to be the cause of the bronchiectasis in this case. This case suggests that airway environmental changes following COVID‐19 may increase the risk of colonisation by common pathogens such as *H. influenzae* . In patients with post‐COVID‐19 bronchiectasis, management focusing on chronic airway infection is important, and strategies to suppress not only acute infection but also long‐term bacterial colonisation and the accompanying chronic inflammation have to be considered.

In conclusion, we experienced a case of newly developed *H. influenzae* infection/colonisation in post‐COVID‐19 bronchiectasis. Airway structural changes following COVID‐19 may increase the risk of chronic airway infection, suggesting the importance of long‐term respiratory follow‐up and bacterial colonisation management. This case highlights new considerations for airway management following COVID‐19, and further research is needed.

## Author Contributions

Conceptualisation: I.O., K.T. Investigation: I.O., Y.O., N.F., S.Y., S.T. Writing original draft preparation: I.O., K.T. Writing review and editing: T.I.1, T.I.2, O.K., K.T. Funding acquisition: K.F. Supervision: K.T.

## Ethics Statement

No ethical committee approval was required for this case report by the Department, because this article does not contain any studies with human participants or animals. Informed consent was obtained from the patient included in this study.

## Consent

The authors declare that written informed consent was obtained for the publication of this manuscript and accompanying images and attest that the form used to obtain consent from the patient complies with the Journal requirements as outlined in the author guidelines.

## Conflicts of Interest

K.F. is an Editorial Board member of Respirology Case Reports and a co‐author of this article. He was excluded from all editorial decision‐making related to the acceptance of this article for publication. The other authors have nothing to declare.

## Data Availability

Data sharing is not applicable to this article as no new data were created or analyzed in this study.
